# Improved hit criteria for DNA local alignment

**DOI:** 10.1186/1471-2105-5-149

**Published:** 2004-10-14

**Authors:** Laurent Noé, Gregory Kucherov

**Affiliations:** 1 LORIA/INRIA-Lorraine, 615, rue du Jardin Botanique, B.P. 101, 54602 Villers-lès-Nancy France

## Abstract

**Background:**

The hit criterion is a key component of heuristic local alignment algorithms. It specifies a class of patterns assumed to witness a potential similarity, and this choice is decisive for the selectivity and sensitivity of the whole method.

**Results:**

In this paper, we propose two ways to improve the hit criterion. First, we define the *group criterion *combining the advantages of the single-seed and double-seed approaches used in existing algorithms. Second, we introduce *transition-constrained seeds *that extend spaced seeds by the possibility of distinguishing transition and transversion mismatches. We provide analytical data as well as experimental results, obtained with the YASS software, supporting both improvements.

**Conclusions:**

Proposed algorithmic ideas allow to obtain a significant gain in sensitivity of similarity search without increase in execution time. The method has been implemented in YASS software available at .

## Background

Sequence alignment is a fundamental problem in Bioinformatics. Despite of a big amount of efforts spent by researchers on designing efficient alignment methods, improving the alignment efficiency remains of primary importance. This is due to the continuously increasing amount of nucleotide sequence data, such as EST and newly sequenced genomic sequences, that need to be compared in order to detect similar regions occurring in them. Those comparisons are done routinely, and therefore need to be done very fast, preferably instantaneously on commonly used computers. On the other hand, they need to be precise, i.e. should report all, or at least a vast majority of interesting similarities that could be relevant in the underlying biological study. The latter requirement for the alignment method, called the *sensitivity*, counterweights the speed requirement, usually directly related to the *selectivity *(called also *specificity*) of the method. The central problem is therefore to improve the trade-off between those opposite requirements.

The Smith-Waterman algorithm [1] provides an exact algorithmic solution to the problem of computing optimal local alignments. However, its quadratic time complexity has motivated the creation of rapid heuristic local alignments tools. A basic idea behind all heuristic algorithms is to focus only on those regions which share some patterns, assumed to witness (or to *hit*) a potential similarity. Those patterns are formed by *seeds *which are small strings (usually up to 25 nucleotides) that appear in both sequences. FASTA [2] and BLAST [3,4] are well-known examples of such methods. BLAST is currently the most commonly used sequence alignment tool, and is a kernel of higher-level search tools, such as PSI-BLAST [4] for instance.

More recently, several new alignment methods have been proposed, such as BLAT [5], PatternHunter [6], LAGAN [7], or BLASTZ [8], to name a few. The improvement brought by all those tools results from new more efficient *hit criteria *that define which pattern shared by two sequences is assumed to witness a potential alignment. Two types of improvements can be distinguished. On the one hand, using two or more closely located smaller seeds instead of one larger seed has been shown to improve the sensitivity/selectivity trade-off [4-6], especially for detecting long similarities. On the other hand, new seed models have been proposed, such as spaced seeds [6], seeds with errors [5], or vector seeds [9].

In this paper, we propose further improvements in both those directions. In the first part (Section *Group hit criterion*), we propose a new flexible and efficiently computable hit criterion, called *group criterion*, combining the advantages of the single-seed ([3]) and multi-seed ([2,4-6]) criteria. In the second part (Section *Generalized seed models*), we propose a new more expressive seed model which extends the spaced seed model of PatternHunter [6] by the possibility of distinguishing transition and transversion mismatches. We show that this allows to obtain a further gain in sensitivity on real genomic sequences, usually rich in transition mutations. Both proposed improvements have been implemented in YASS software [10], used in the experimental part of this work.

## Results

### Group hit criterion

The first preparatory step of most heuristic alignment algorithms consists of constructing a hash table of all *seeds *occurring in the input sequences. In this section, we assume that a seed of *weight k *is a word consisting of *k *contiguous nucleotides (*k*-word), more general notions of seed will be considered in the next Section.

In the simplest case, implemented in the early version of BLAST [3], an individual seed occurring in both sequences acts as a *hit *of a potential alignment. It triggers the *X-drop *algorithm trying to extend the seed to a so-called High-scoring Segment Pair (HSP), used then to obtain a larger final alignment. Gapped BLAST [4] proposes a *double-seed *criterion that defines a hit as two non-overlapping seeds occurring at the same dotplot diagonal within a fixed-size window. This allows to considerably increase the selectivity with respect to the single-seed approach, and at the same time to preserve, and even to improve, the sensitivity on large similarities. On the other hand, Gapped BLAST is less sensitive on short and middle-size similarities of weak score. (We will show this more formally at the end of this Section.) Most of the existing alignment programs [5,6] use either a single-seed or a double-seed hit criterion.

Here we propose a new flexible hit criterion defining a hit as a group containing an arbitrary number of possibly overlapping seeds, with an additional constraint on the minimal number of matching nucleotides. The seeds of the same group are assumed to belong to the same similarity, and therefore should be proximate to each other. In contrast to other multi-seed hit criteria [4-6], we don't require seeds to occur at the same dotplot diagonal but at close diagonals, to account for possible indels. The possible placement of seeds is controlled by parameters computed according to statistical models that we describe now.

#### Group criterion

A hit criterion defines a pattern which is considered as an evidence of a potential similarity. Every time this pattern is found, its extension is triggered to compute a potential larger alignment. The extension is usually done via a dynamic programming algorithm and is a costly step. The hit criterion should be *selective *enough to avoid spurious extensions and, on the other hand, should be *sensitive *to detect as many relevant similarities as possible.

The hit criterion we propose is based on a *group of seeds *verifying conditions (1), (2) (see Section *Methods*). By the considered statistical analysis, this ensures a good sensitivity. However, many groups will contain a single seed or two strongly overlapping seeds, that either belong to a similarity with a low score, or do not belong to any similarity at all (i.e. don't belong to an alignment with a sufficiently high score). To cope with this problem, we introduce an additional criterion that selects groups that will be actually extended. The criterion, called *group criterion*, is based on the *group size *defined as the *minimal number of matching individual nucleotides in all seeds of the group*. The group size can be seen as a parameter specifying the maximal overlap of the seeds of a group. For example, if the group size is *k *+ 1, then no constraint on the overlap is imposed, i.e. any group containing two distinct seeds forms a hit. If the group size is 2*k*, then the group must contain at least two non-overlapping seeds, etc.

Allowing overlapping seeds is an important point that brings a flexibility to our method. Note that other popular multi-seed methods [4,5] consider only non-overlapped seeds. Allowing overlapped seeds and controlling the overlap with the group size parameter offers a trade-off between a single-seed and a multi-seed strategies. This increases the sensitivity of the usual multi-seed approach without provoking a tangible increase in the number of useless extensions. In the next section, we will provide quantitative measures comparing the sensitivity of the YASS group criterion with BLAST and Gapped BLAST.

#### Some comparative and experimental data

In this section, we adopt the following experimental setup to estimate the sensitivity of the YASS group criterion compared to other methods. We first set a match/mismatch scoring system, here fixed to +1/-3 (default NCBI-BLAST values). The main assumption is that the sensitivity is estimated as the probability of hitting a random *gapless alignments of a fixed score*. Moreover, to make this model yet more close to reality, only *homogeneous alignments *are considered, i.e. alignments that don't contain proper sub-alignments of bigger score (see [11]). For a given alignment length, all homogeneous alignments are assumed to have an equal probability to occur.

**Figure 1:**
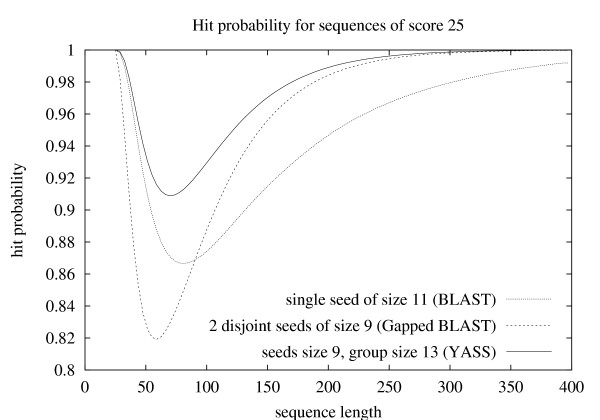
**Hit Probability. **Hit probability as a function of length of fixed-score alignments

In this setting, we computed the hit probability of a single-seed criterion with seed weight 11 (default for BLAST) and compared it with multi-seed criteria of Gapped BLAST and YASS for seed weight 9 (default for Gapped BLAST). For YASS, the group size was set to 13. Figure 1 shows the probability graphs for alignment score 25.

Comparing BLAST and Gapped BLAST, the former is more sensitive on short similarities (having higher identity rate), while the latter is more sensitive on longer similarities, in which two close non-overlapping runs of 9 matches are more likely to occur than one run of 11 matches. The YASS group criterion combines the advantages of both: it is more sensitive than the single-seed criterion even for short similarities, and than the non-overlapping double-seed criterion for large similarities (Figure 1).

Note, however, that for the chosen parameters, the YASS criterion is slightly less selective than that of Gapped BLAST as it includes any two non-overlapping seeds but also includes pairs of seeds overlapped by at most 5 bp. The selectivity can be estimated by the probability of a hit at a given position in a random uniform Bernoulli sequence (see [5]). For YASS, this probability is 2.1·10^-8^, which improves that of BLAST (2.4·10^-7^) by more than ten. For Gapped BLAST, this probability is 7.3·10^-9^. To make an accurate sensitivity comparaison of YASS and Gapped BLAST, parameters should be set so that both algorithms have the same selectivity level.

To compare the sensitivity of YASS and Gapped BLAST for an equal selectivity level, we chose a parameter configuration such that both algorithms have the same estimated selectivity (10^-6^). This is achieved with seed weight 8 for Gapped BLAST and group size 11 for YASS (while keeping seed weight 9). In this configuration, and for sequences of score 25, YASS turns out to be considerably more sensitive on sequences up to 80 bp and is practically as sensitive as Gapped BLAST on longer sequences (data not shown). At the same time, YASS is more time efficient in this case, as Gapped BLAST tends to compute more spurious individual seeds that are not followed by a second hit, which takes a considerable part of the execution time. This is because the YASS seed is larger by one nucleotide, and the number of spurious individual seeds computed at the first step is then divided by 4 on large sequences.

Compared to the single-seed criterion of BLAST, the YASS group criterion is both more selective (group size 13 vs single-seed size 11) and more sensitive *for all alignment lengths*, as soon as the alignment score is 25 or more. If the score becomes smaller, both criteria yield an unacceptably low sensitivity, and the seed weight has then to be decreased to detect those similarities.

Finally, we point out another experiment we made to bring more evidence that the group criterion captures a good sensitivity/selectivity trade-off. We monitored the partition of the execution time between the formation of groups and their extension by dynamic programming (data not shown). It appeared that YASS spends approximately equal time on each of the two stages, which gives a good indication that it provides an optimal distribution between the two complementary parts of the work.

### Generalized seed models

So far, we defined seeds as *k*-words, i.e. short strings of *contiguous *nucleotides. Recently, it has been understood that using *spaced seeds *allows to considerably improve the sensitivity. A spaced seed is formed by two words, one from each input sequence, that match at positions specified by a fixed *pattern *– a word over symbols # and _ interpreted as a match and a don't care symbol respectively. For example, pattern ##_# specifies that the first, second and fourth positions must match and the third one may contain a mismatch.

PatternHunter [6] was the first method that used carefully designed spaced seeds to improve the sensitivity of DNA local alignment. In [12], spaced seeds have been shown to improve the efficiency of *lossless *filtration for approximate pattern matching, namely for the problem of detecting all matches of a string of length *m *with *q *possible substitution errors (an (*m*, *q*)-problem). The use of some specific spaced seeds for this problem was proposed earlier in [13]. Yet earlier, random spaced seeds were used in FLASH software [14] to cover sequence similarities, and the sensitivity of this approach was recently studied in [15].

The advent of spaced seeds raised new questions: How to choose a good seed for a local alignment algorithm? How to make a precise estimation of the seed goodness, or more generally, of a seed-based local alignment method? In [16], a dynamic programming algorithm was proposed to measure the hit probability of a seed on alignments modeled by a Bernoulli model. In the lossless case, an analogous algorithm that allows to test the seed completeness for an (*m*, *q*)-problem was proposed in [12]. The algorithm of [16] has been extended in [17] for hidden Markov models on order to design spaced seeds for comparing homologous coding regions. Another method based on finite automata was proposed in [18]. A complementary approach to estimate the seed sensitivity is proposed in [11]. Papers [19,20] present a probabilistic analysis of spaced seeds, as well as experimental results based on the Bernoulli alignment model.

Other extensions of the contiguous seed model have been proposed. BLAT [5] uses contiguous seeds but allows one error at any of its positions. This strategy is refined in BLASTZ [8] that uses spaced seeds and allows one transition substitution at any of match positions. An extension, proposed in [9], enriches the PatternHunter spaced seeds model with a scoring system to define a hit.

Here we propose a new *transition-constrained seed *model. Its idea is based on the well-known feature of genomic sequences that transition mutations (nucleotide substitutions between purins or between pyrimidins) occur relatively more often than transversions (other substitutions). While in the uniform Bernoulli sequence transitions are twice less frequent than transversions, in real genomic sequences this ratio is often inverted. For example, matrices computed in [21] on mouse and human sequences imply that the transition/transversion rate (hereafter *ti/tv*) is greater than one on average. Transitions are much more frequent than transversions in coding sequences, as most of silent mutations are transitions. *ti/tv *ratio is usually greater for related species, as well as for specific DNA (mitochondrial DNA for example).

Transition-constrained seeds are defined on the ternary alphabet {#, @, _}, where @ stands for a match or a transition mismatch (A ↔ G, C ↔ T), and # and _ have the same meaning as for spaced seeds. The *weight of a transition-constrained seed *is defined as the sum of the number of #'s plus half the number of @'s. This is because a transition carries one bit of information while a match carries two bits.

Note that using transition-constrained seeds is perfectly compatible with the group criterion described in Section *Group criterion*. The only non-trivial algorithmic issue raised by this combination is how to efficiently compute the group size during the formation of groups out of found seeds. In YASS, this is done via a special finite automaton resulting from the preprocessing of the input seed.

#### Transition-constrained seeds for Bernoulli alignment model

To estimate the detection capacity of transition-constrained seeds, we first use the Bernoulli alignment model, as done in [6,19,20]. We model a gapless alignment by a Bernoulli sequence over the ternary match/transition/transversion alphabet with the match probability 0.7 and the probabilities of transition/transversion varying according to the *ti/tv *ratio. The sequence length is set to 64, a typical length of a gapless fragment in DNA alignments. We are interested in seed weights between 9 and 11, as they represent a good sensitivity/selectivity compromise.

Table 1 compares the sensitivity of the best spaced seeds of weight 9, 10 and 11, reported in [20], with some transition-constrained seeds, assuming that transitions and transversions occur with equal probability 0.15. The transition-constrained seeds have been obtained using a stepwise Monte-Carlo search, and the probabilities have been computed with an algorithm equivalent to that of [16]. The table shows that transition-constrained seeds are more sensitive with respect to this model.

**Figure 2:**
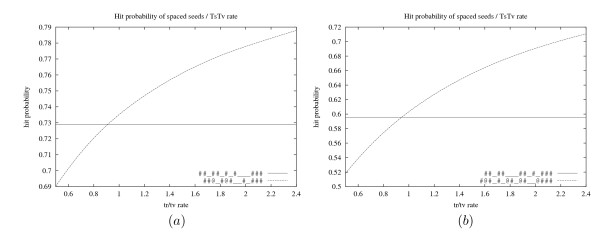
**Seed Probability. **Hit probability of seed models on Bernoulli sequences as a function on *ti/tv *ratio

A natural question is the efficiency of transition-constrained seeds depending on the *ti/tv *ratio. This is shown in Figure 2. The left and right plots correspond to the seeds from Table 1 of weight 9 and 10 respectively. The plots show that the sensitivity of transition-constrained seeds greatly increases when the *ti/tv *ratio is over 1, which is typically the case for real genomic sequences.

#### Transition-constrained seeds for Markov alignment model

Homologous coding sequences, when aligned, usually show a regular distribution of errors due to protein coding constraints. In particular, transitions often occur at the third codon position, as these mutations are almost always silent for the resulting protein. Markov models provide a standard modeling tool to capture such local dependencies. In the context of seed design, papers [16-18] proposed methods to compute the hit probability of spaced seeds with respect to gapless alignments specified by (Hidden) Markov models. To test whether using transition-constrained seeds remains beneficial for alignments specified by Markov models, we constructed a Markov model of order 5 out of a large mixed sample of about 100 000 crossed alignments of genomic sequences of distantly related species (*Neisseria Meningitidis, S. Cerevisiae, Human X chromosome, Drosophila*). The alignments were computed with different seeds of small weight, to avoid a possible bias caused by a specific alignment method. We then designed optimal spaced and transition-constrained seeds of weight 9–11 with respect to this Markov model. Table 2 shows the results of this computation providing evidence that transition-constrained seeds increase the sensitivity with respect to this Markov model too.

### Experiments

#### Seed experiments

In order to test the detection performance of transition-constrained seeds on real genomic data, we made experiments on full chromosomic sequences of *S. Cerevisiae *(chromosomes IV, V, IX, XVI) and *Neisseria meningitidis *(strains MC58 and Z2491). The experiments were made with our YASS software [10] that admits user-defined transition-constrained seeds and implements the group criterion described in Section *Group criterion*. The experiments used seeds of weight 9 and 11, obtained on Bernoulli and Markov models (reported in Tables 1 and 2). The search was done using group size 10 and 12 respectively for seed weight 9 and 11 (option -s of YASS). This means that at least two distinct seeds were required to trigger the extension, with no additional constraint on their overlap, which is equivalent to the double-seed criterion of PatternHunter. The scoring system used was +1/-1 for match/mismatch and -5/-1 for gap opening/extension. Both strands of input chromosomes has been processed in each experiment (-r 2 option of YASS).

For each comparison, we counted the number of computed alignments with E-value smaller than 10^-3^. Table 3 reports some typical results of this experiment. They confirm that using transition-constrained seeds does increase the search sensitivity. A side (non-surprising) observation is that, in all tests, the seed designed on the Markov model turns out to be more efficient than the one designed on the Bernoulli model. Note that the similarity search can be further improved by using transition-specific scoring matrices (for example, PAM Transition/Transversion matrices or matrices designed for specific comparisons [21]) rather than uniform matches/mismatch matrices, and transition-constrained spaced seeds are more likely to detect alignments highly scored by those matrices.

Another advantage of transition-constrained seeds comes from the fact that they are more robust with respect to the GC/AT composition bias of the genome. This is because purins and pyrimidins remain balanced in GC- or AT-rich genomes, and one match carries less information (is more likely to occur "by chance") than two match-or-transition pairs.

#### Program experiments

A series of comparative tests has been carried out to compare the sensitivity with traditional approaches. Several complete bacterial genomes ranging from 3 to 5 Mb have been processed against each other using both YASS and the b12seq programs (NCBI BLAST package 2.2.6.). The tests used the scoring system +1/-1 for match/mismatch and -5/-1 for gap opening/extension. The threshold E-value for the output was set to 10 (default BLAST value), and the sequence filtering was disabled. YASS was run with its default seed pattern #@#__##__#_##@# of weight 9 which provides a good compromise in detecting similarities of both coding and non-coding sequences.

For each test, the number of alignments with E-value less than 10^-6 ^found by each program, and the number of exclusive alignments were reported. By "exclusive alignment", we mean every alignment of E-value less than 10^-6 ^that does not share a common part (do not overlap on both sequences) with any alignment found by the other program. To take into account a possible bias caused by splitting alignments into smaller ones (X-drop effect), we also computed the total length of exclusive alignments, found by each program.

Experiments are summarized in Table 4 and show that within a generally smaller execution time, YASS detects more exclusive similarities that cover about twice the overall length of those found by b12seq. The gain in execution time increases when the sequence length gets larger.

## Conclusions

In this paper, we introduced two independent improvements of hit criteria for DNA local alignment. The *group criterion*, based on statistical DNA sequence models, combines the advantages of both single-seed and double-seed criteria. *Transition-constrained seeds *account for specificities of real DNA sequences and allow to further increase the search sensitivity with respect to spaced seeds. Both options have been implemented in YASS software available at .

Transition-constrained seeds could be further extended using the idea of vector seeds [9], i.e. by assigning weights to each seed position, but also to each type of mutation. This would give a more general mechanism to account for the information brought by different mutations. But the model is also more flexible, an thus involves a larger search space to design seeds.

Another new direction for further improving the efficiency is a simultaneous use of several seed patterns [22-24], complementing the sensitivity of each other. However, designing such families is also hard problem, due to the involved search space.

## Methods

### Statistical analysis

We first introduce some notations used in this section. Let *S*_1 _and *S*_2 _be the input sequences of length *m *and *n *respectively. Each of them can be considered as a succession of *m *- *k *+ 1 (respectively *n *- *k *+ 1) substrings of length *k*, called *k-words*. If a *k*-word of *S*_1 _matches another *k*-word of *S*_2_, i.e. *S*_1_[*i*..*i *+ *k *- 1] = *S*_2_[*j*..*j *+ *k *- 1] for some *i *≤ *m *and *j *≤ *n*, then these two *k-*words form a *seed *denoted <*i*, *j*>. Two functions on seeds are considered: For a seed <*i*, *j*>, the *seed diagonal d*(<*i*, *j*>) is *m *+ *j *- *i*. It can be seen as the distance between the *k*-words *S*_1_[*i*..*i *+ *k *- 1] and *S*_2_[*j*..*j *+ *k *- 1] if *S*_2 _is concatenated to *S*_1_, For two seeds <*i*_1_, *j*_1_> and <*i*_2_, *j*_2_>, where *i*_1 _<*i*_2 _and *j*_1 _<*j*_2_, the *inter-seed *distance *D*(<*i*_1_, *j*_1_>, <*i*_2_, *j*_2_>) is the maximum between |*i*_2 _- *i*_1_| and |*j*_2 _- *j*_1_|. The problem considered in this Section is to derive conditions under which two seeds are likely to be a part of *the same *alignment, and therefore should be grouped together. More precisely, we want to be able to compute parameters *ρ *and *δ *such that two *seeds *<*i*_1_, *j*_1_> and <*i*_2_, *j*_2_> have a probability (1 - *ε*) to belong to the same similarity iff

*D*(<*i*_1_, *j*_1_>, <*i*_2_, *j*_2_>) ≤ *ρ*,     (1)

|*d*(<*i*_1_, *j*_1_>) - *d*(<*i*_2_, *j*_2_>)| ≤ *δ*.     (2)

The first inter-seed condition insures that the seeds are close enough to each other. The second seed diagonal condition requires that in both seeds, the two *k*-words occur at *close diagonals*.

We now describe statistical models used to compute parameters *ρ *and *δ*.

#### Bounding the inter-seed distance

Consider two homologous DNA sequences that stem from a duplication of a common ancestor sequence, followed by independent individual substitution events. Under this assumption, the two sequences have an equal length and their alignment is a sequence of matched and mismatched pairs of nucleotides. We model this alignment by a Bernoulli sequence with the probability *p *for a match and (1 - *p*) for a mismatch. To estimate the inter-seed shift *D*_*k*_, we have to estimate the distance between the starts of two *successive runs of at least k matches *in the Bernoulli sequence. It obeys the geometric distribution of order *k *called the *Waiting time distribution *[25,26]:


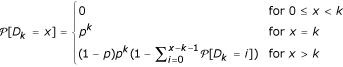


Using this formula, we compute *ρ *such that the probability 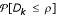 is (1 - *ε*) for some small *ε*.

Note that the Waiting time distribution allows us to estimate another useful parameter: the number of runs of matches of length at least *k *inside a Bernoulli sequence of length *x*. In a Bernoulli sequence of length *x*, the probability of the event *I*_*p*,*x*,*r *_of having exactly *r non-overlapping *runs of matches of length at least *k *is given by the following recursive formula:


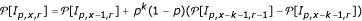


This gives the probability of having exactly *r non-overlapping *seeds of length at least *k *inside a repeat of size *x*. The recurrence starts with *r *= 0, in which case 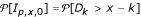 and is computed through the Waiting time distribution.

The distribution 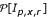 allows us to infer a lower bound on the number of non-overlapping seeds expected to be found inside a similarity region. In particular, we will use this bound as a first estimate of the group criterion introduced later.

#### Bounding the seed diagonal variation

*Indels *(nucleotide insertions/deletions) are responsible for a diagonal shift of seeds viewed on a dotplot matrix. In other words, they introduce a possible difference between *d*(<*i*_1_, *j*_1_>) and *d*(<*i*_2_, *j*_2_>). To estimate a typical shift size, we use a method similar to the one proposed in [26] for the search of tandem repeats.

Assume that an indel of an individual nucleotide occurs with an equal probability *q *at each of *l *nucleotides separating two consecutive seeds. Under this assumption, estimating the diagonal shift produced by indels is done through a discrete one-dimensional *random walk *model, where the probability of moving left or right is equal to *q*, and the probability of staying in place is 1 - 2*q*. Our goal is to bound, with a given probability, the deviation from the starting point.

The probability of ending the random walk at position *i *after *l *steps is given by the following sum:


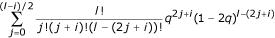


A direct computation of multi-monomial coefficients quickly leads to a memory overflow, and to circumvent this, we use a technique based on generating functions. Consider the function 
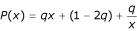
 and consider the power *P*^*l*^(*x*) = *a*_*l*_.*x*^*l *^+…+ *a*_-*l*_.*x*^-*l*^. Then the coefficient *a*_*i *_computes precisely the above formula, and therefore gives the probability of ending the random walk at position *i *after *l *steps. We then have to sum up coefficients *a*_*i *_for *i *= 0,1, -1, 2, -2,..., *l*, -*l *until we reach a given threshold probability (1 - *ε*). The obtained value *l *is then taken as the parameter *δ *used to bound the maximal diagonal shift between two seeds.

## Figures and Tables

**Table 1:** Bernouilli Model Hit probability of seeds on Bernoulli sequences of length 64 with match probability 0.7 and transition/transversion probabilities 0.15

weight	spaced seed	hit proba	transition-constrained seed	hit proba
9	*B*_9 _= ##_##_#_#___###	0.7291	*B*_9_^*tr *^= ##@_#@#__#_###	0.7366
10	B_10 _= ##_##___##_#_###	0.5957	*B*_10_^*tr *^= #@#_#_@#_@#__@###	0.6056
11	*B*_11 _= ###_#__#_#__##_###	0.4671	*B*_11_^*tr *^= #@#_#@__##_#_@@##	0.4784

**Table 2:** Markov Model Hit probability of seeds on a Markov model of order 5 trained on a large mixed sample of cross-species alignments

weight	spaced seed	hit proba	transition-constrained seed	hit proba
9	*M*_9 _= ##_##_##_###	0.822	*M*_9_^*tr *^= ##@##_##@##	0.845
10	*M*_10 _= ##_##_##___##_##	0.716	*M*_10_^*tr *^= #@@##_##_##@#@	0.746
11	*M*_11 _= ##_##_##_##_###	0.603	*M*_11_^*tr *^= ##@##_##_##@##	0.632

**Table 3:** Seed experiments. Number of high-scoring similarities found with different seed patterns

sequences	*B*_9_	*B*_9_^*tr*^	*B*_11_	*B*_11_^*tr*^	*M*_9_	*M*_9_^*tr*^	*M*_11_	*M*_11_^*tr*^
IX/V	323	336	275	279	312	325	274	293
IX/XVI	342	354	271	280	349	357	280	295
XVI/IV	1314	1361	1124	1172	1309	1348	1180	1235
MC58/Z2491	361896	380028	341113	364792	385444	392164	359348	366759

**Table 4:** Comparative Tests. Comparative tests of YASS vs b12seq (NCBI BLAST 2.2.6). Reported execution times have been obtained on a Pentium IV 2.4 GHz computer.

sequence 1		sequence 2		time (sec)		# align.		# ex. align.		ex. align. length	
name	size	name	size	Y.	B.	Y.	B.	Y.	B.	Y.	B.
*S.sp.*	3.6	*M.t.*	4.4	122	148	494	310	130	27	29145	7970
*S.sp.*	3.6	*C.g.*	3.3	161	163	578	369	168	63	37310	30138
*S.sp.*	3.6	*Y.p.*	4.6	156	253	901	617	186	54	39354	19994
*S.sp.*	3.6	*V.p.*	3.3	164	167	940	465	349	60	65788	28883
*M.t.*	4.4	*C.g.*	3.3	211	542	1851	1265	397	160	102103	80012
*M.t.*	4.4	*Y.p.*	4.6	168	255	738	515	197	86	44348	23361
*M.t.*	4.4	*V.p.*	3.3	72	69	498	295	171	30	36474	12021
*C.g.*	3.3	*Y.p.*	4.6	130	161	962	640	186	45	34538	11277
*C.g.*	3.3	*V.p.*	3.3	95	93	1109	687	197	72	42009	21575
*Y.p.*	4.6	*V.p.*	3.3	149	217	2900	1953	622	264	186585	110352

*C.g*: *Corynebacterium glutamicum ATCC 13032*,

*M.t*: *Mycobacterium tuberculosis (CDC1551)*,

*S.sp.*: *Synechocystis sp. PCC 6803*,

*S.sp.*: *Vibrio parahaemolyticus RIMD 2210633 chr I*,

*Y.p.*: *Yersinia pestis KIM*
